# Reference Genes to Study Herbicide Stress Response in *Lolium* sp.: Up-Regulation of P450 Genes in Plants Resistant to Acetolactate-Synthase Inhibitors

**DOI:** 10.1371/journal.pone.0063576

**Published:** 2013-05-16

**Authors:** Arnaud Duhoux, Christophe Délye

**Affiliations:** INRA, UMR1347 Agroécologie, Dijon, France; Kyushu Institute of Technology, Japan

## Abstract

Variation in the expression of numerous genes is at the basis of plant response to environmental stresses. Non-target-site-based resistance to herbicides (NTSR), the major threat to grass weed chemical control, is governed by a subset of the genes involved in herbicide stress response. Quantitative PCR assays allowing reliable comparison of gene expression are thus key to identify genes governing NTSR. This work aimed at identifying a set of reference genes with a stable expression to be used as an internal standard for the normalisation of quantitative PCR data in studies investigating NTSR to herbicides inhibiting acetolactate synthase (ALS) in the major grass weed *Lolium* sp. Gene expression stability was assessed in plants resistant or sensitive to two ALS inhibitors, subjected or not to herbicide stress. Using three complementary approaches implemented in the programs BestKeeper, NormFinder and geNorm, cap-binding protein, glyceraldehyde-3-phosphate-dehydrogenase and ubiquitin were identified as the most suitable reference genes. This reference gene set can probably be used to study herbicide response in other weed species. It was used to compare the expression of the genes encoding two herbicide target enzymes (ALS and acetyl-coenzyme A carboxylase) and five cytochromes P450 (CYP) with potential herbicide-degrading activity between plants resistant or sensitive to ALS inhibitors. Overall, herbicide application enhanced *CYP* gene expression. Constitutive up-regulation of all *CYP* genes observed in resistant plants compared to sensitive plants suggested enhanced secondary metabolism in the resistant plants. Comprehensive transcriptome studies associated to gene expression analyses using the reference gene set validated here are required to unravel NTSR genetic determinants.

## Introduction

Plant response to environmental stresses is mediated by the regulation of gene expression. A major abiotic stress encountered by arable weeds infesting agricultural fields is herbicide applications. Herbicide applications therefore trigger stress response pathways in weed plants [1]. Due to inherent intraspecific genetic variation, these pathways can differ among individual weed plants. In some plants, some of the stress response pathways triggered by herbicide applications can enable plants to survive herbicide applications. These particular pathways are at the basis of non-target-site based resistance (NTSR) to herbicides, an adaptive response [1]. NTSR is the major cause for herbicide resistance in grass weeds, and is thus agronomically and economically very important [1]. As a part of plant stress response pathways, NTSR is under a complex genetic control that is still poorly understood, but involves changes in the regulation of a range of genes in resistant plants compared to sensitive plants. In particular, an increase in glutathione-S-transferase, cytochrome P450 (CYP) or glycosyl-transferase enzyme activities leading to an acceleration of herbicide degradation in herbicide-resistant weed plants has often been observed, but hardly any data is available regarding the genes involved [1]. Yet, identifying NTSR genes is crucial for understanding, diagnosing and managing herbicide resistance. As NTSR seems largely endowed by differences in gene expression between resistant and sensitive plants, identifying NTSR genes requires to reliably be able to quantify differences in gene expression.

Quantitative reverse transcription-polymerase chain reaction (RT-qPCR) is the most accurate tool to date to accurately determine differences in gene expression [2]. For this purpose, it is necessary to normalise qPCR data using a set of reference genes with a constant expression level in the system studied [2], [3]. In plants, suitable reference genes have mostly been identified in species with associated genomic resources, such as crop species (e.g. [4]), or model species (*Arabidopsis thaliana* and *Brachypodium distachyon*; e.g. [5], [6]). Few sets of reference genes have been validated for weeds, because most weeds are species without associated genomic resources. Extrapolation from other species, even in closely related taxa, can be tricky: several reports have shown that the expression of many widely used reference genes can vary considerably with the experimental conditions, tissues and species [7], [8]. As a consequence, no “universal” reference genes have been identified to date.

To date, only one set of reference genes has been validated under herbicide stress in a study considering the grass weed *Alopecurus myosuroides* and herbicides inhibiting acetyl-CoA carboxylase (ACCase) [9]. Here, we considered the two herbicides inhibiting acetolactate-synthase (ALS) that are most broadly used against the grass weed *Lolium sp.* (rye-grass). *Lolium sp*. is one of the world’s worst weeds, with a major economic and agronomic negative impact [10]. It is ranked first among the most herbicide-resistance-prone weeds worldwide [10]. ALS is a key enzyme in the biosynthesis of branched amino-acids. ALS inhibitors are the second most broadly used herbicide class worldwide, and are also the herbicide class that is most prone to select for resistance in weeds [10], [11]. NTSR to herbicides is frequently observed in *Lolium* sp. [12]. While CYP activity has been shown to play a role in NTSR of *Lolium sp*. to ALS inhibitors [12], none of the genes involved has been identified to date. This is in part due to the absence of relevant reference genes enabling reliable measurement of gene expression levels under herbicide stress in *Lolium sp*.

The first aim of this study was therefore to validate a set of reference genes with a stable expression under ALS-inhibiting herbicide stress in *Lolium sp*. This set was used for a secondary objective: assessing the effect of ALS inhibitor application on the expression of two genes encoding herbicide target proteins and of five genes encoding CYPs with potential herbicide-degrading activity in plants resistant or sensitive to ALS inhibitors, in order to identify candidate NTSR genes.

## Materials and Methods

### *Lolium* sp. Populations

Seeds of four distinct *Lolium sp.* populations (RG08-994, RG08-914, RG08-068 and RG07-043) were collected in French fields where control of *Lolium sp*. using ALS inhibitors failed. No specific permits were required for the collection of *Lolium sp*. seeds. *Lolium sp*. is not an endangered or protected species, and can freely be collected in agricultural fields by governmental institutions such as INRA. Seedlings from each population were grown in individual 2L-pots in a glasshouse at 22°C/18°C day/night with 14-hour photoperiod. To ensure resistant plants were resistant because of NTSR, the occurrence of mutations known to endow herbicide resistance within the gene encoding ALS was excluded by genotyping all seedlings at the *ALS* locus as described [13] prior to herbicide application.

When each plant had developed at least a dozen tillers, the individual tillers were separated and transplanted into individual pots to obtain individual one-tiller plants. The one-tiller plants issued from a same plant were clones, i.e., genetically identical plants at the same growth stage (3–4 leaves). This allowed to use a given plant in different experimental modalities.

### Plant Material Production for the Validation of a Reference Gene Set

A batch of samples was produced to assess the stability of expression of candidate reference genes. A time-course experiment consisting of six modalities was conducted for each herbicide studied. Modalities were: before treatment (BT), 2 hours after treatment (2HAT), 6 hours after treatment (6HAT), 24 hours after treatment (24HAT), unsprayed control and sprayed control. Two clones were used par plant and per modality, i.e., a total of 12 clones par plant studied. A sample consisted of the above-ground part of the two clones used for a given plant and a given modality that was cut, immediately frozen in liquid nitrogen, and stored at −80°C prior to RNA extraction. The two ALS-inhibiting herbicides most frequently sprayed against *Lolium sp*. were used for treatment: a mixture of two sulfonylureas (iodosulfuron+mesosulfuron; Archipel WG; 3% w/w iodosulfuron and mesosulfuron; Bayer CropScience, applied at the recommended field rate: 250 g formulated herbicide per ha) and a triazolopyrimidine (pyroxsulam; Abak; 7.5% w/w pyroxsulam; Dow Agroscience, applied at the recommended field rate: 250 g formulated herbicide per ha). An adjuvant enhancing the herbicide penetration into the plant tissues was added to the mixture as recommended (Actirob B; Bayer CropScience, applied at the recommended field rate: 1 L per ha). Herbicide application using a custom-built, single-nozzle sprayer was as described [9]. At least two clones from a reference herbicide-sensitive plant were included in each spraying experiment to check herbicide application efficacy.

The sprayed control modality was used to determine the phenotype (resistant or sensitive) of the source plant by observing clone survival four weeks after herbicide application. If the two clones were hardly damaged and extended new, green leaves, the source plant was considered resistant. If the two clones were dead, the source plant was considered sensitive. If the two clones were damaged but extended new, green leaves, the source plant was considered moderately resistant. The samplings produced using (iodosulfuron+mesosulfuron) and pyroxsulam will be referred to as sampling IM1 and sampling P1, respectively (Table 1).

**Table 1:** RNA samples used to assess the stability of candidate reference genes (samplings IM1 and P1).

Sampling IM1. Herbicide: (iodosulfuron+mesosulfuron)										
Herbicide application^1^	Populations									
	RG08-994		RG08-914		RG08-068		RG07-043		Total	
	S^2^	R	S	R	S	R	S	R	S	R
BT	2	1	1	3	1	1	1	0	5	5
2HAT	2	1	1	3	1	1	1	0	5	5
6HAT	2	2	1	3	1	0	1	0	5	5
24HAT	2	2	1	1	1	2	1	0	5	5
Total	8	6	4	10	4	4	4	0	20	20
**Sampling P1. Herbicide: pyroxsulam.**										
**Herbicide application** 1	**Populations**									
	**RG08-994**		**RG08-914**		**RG08-068**		**RG07-043**		**Total**	
	**S** 2	**R**	**S**	**R**	**S**	**R**	**S**	**R**	**S**	**R**
BT	0	1	3	2	0	2	2	0	5	5
2HAT	0	1	3	2	0	1	2	1	5	5
6HAT	2	2	2	2	0	1	1	0	5	5
24HAT	0	1	3	1	0	3	2	0	5	5
Total	2	5	11	7	0	7	7	1	20	20

1 BT, before treatment; xHAT, x hours after treatment.

2 S, sensitive; R, resistant.

### Plant Material Production to Assess the Effect of Herbicide Application on the Expression of Seven Genes of Interest

A second batch of samples was produced to measure the expression of genes of interest in the presence or absence of herbicide. For this purpose, a second time-course experiment was conducted as before for both herbicides studied. The modalities consisted of clones collected before treatment (BT) and 24 hours after treatment (24HAT), unsprayed control and sprayed control. The samplings produced using (iodosulfuron+mesosulfuron) and pyroxsulam in this experiment will be referred to as sampling IM2 and sampling P2, respectively (Table 2).

**Table 2:** Number of plants in the samplings used for target gene expression quantification BT and 24HAT (samplings IM2 and P2).

Sampling	Populations										
	RG08-068		RG08-994			RG08-914			RG07-043		
	S^2^	R	S	R	r	S	R	r		S	R
IM2^1^	5	5	4	4	1	3	2	1		3	0
P2^1^	0	0	0	0	0	5	2	1		0	0

1 IM2, sprayed with iodosulfuron+mesosulfuron; P2, sprayed with pyroxsulam.

2 S, sensitive; R, resistant, r, moderately resistant. All plants were analysed before treatment (BT) and 24 hours after treatment (24HAT).

### cDNA Synthesis

Total RNA extraction and DNA contamination removal were performed using the RNeasy plant mini kit and the RNase free DNase set (Qiagen, Courtaboeuf, France) according to the manufacturer’s instructions. Nucleic acid concentration was measured at 260 nm using a NanoDrop ND-1000 spectrophotometer (LABTECH, Luton, UK). RNA quality was considered adequate for A_260_/A_280_ and A_260_/A_230_ absorption ratio values between 1.8 and 2.2, and 2 and 2.2, respectively. cDNA synthesis was performed from 5 µg total RNA for each sample using the Masterscript RT-PCR System (5 PRIME, Hamburg, Germany). For each RNA sample used, two independent reverse-transcription reactions were performed to obtain technical replicates, as recommended [3].

### Primer Design and PCR Conditions

The candidate reference genes tested were reference genes which expression had been proven stable under various stresses in *Lolium* sp. [14], [15] or in other grasses [4], [16], [17]. Primer sequences are given in Table 3. They were designed using the available genes in *Lolium perenne*, *Lolium rigidum* or *Lolium temulentum* deposited in GenBank/EMBL. The web interface Primer3Plus [18] was used to design primers using a primer length of 21±3 nucleotides, a melting temperature (Tm) of 60°C ±3°C, a guanine-cytosine content between 40% and 60% and an expected amplicon size between 90 and 150 base pairs. Primers targeting *TUB* and *GAPDH* were designed to amplify an intron-containing amplicon to enable the detection of gDNA contaminations.

**Table 3:** Candidate reference genes tested and primer information.

Code^1^(GenBank accession)	Primer sequence (5′-3′)^2^	Ampliconlength (bp)	Tm (°C)	PCR efficiency (%)^3^	Regressioncoefficient^3^	Cq^4^
*TUB*	(F) ATACAATGCCACTCTCTCAGTC	140	60	IM1∶101	0,996	23.6
(AY742902)	(R) GAGATGAGATGGTTCAAATCAC			P1∶101	0.999	
*CAP*	(F) CTCCAGGGAAGATGCTGAAG	95	57	IM1∶96	0.996	25.7
(EU328530)	(R) CTTGAAAGCCCCAATCAAAA			P1∶96	0.993	
*EF1*	(F) CACTGGTCACCTGATCTACAA	100	60	IM1∶102	0.990	20.1
(EU168438)	(R) GTACTTGAAGGACCTCTTGTTCA			P1∶93	0.999	
*GAPDH*	(F) AGGTTATCAATGACAAGTTTGG	83	60	IM1∶103	0.999	20.8
(EU328533)	(R) ATCAACAGTCTTCTGGGTAGC			P1∶104	0.994	
*RUB*	(F) GGAGTATGAAACCAAGGATACTG	123	57	IM1∶97	0.993	16.8
(HM850132)	(R) GTTGTCCATGTACCAGTAGAAGA			P1∶99	0.990	
*UBQ*	(F) CAAGAAGAAGACGTACACCAAG	85	57	IM1∶95	0.997	22.2
(EF470423)	(R) GACCTTGTAGAACTGGAGGAG			P1∶101	0.990	
*18S*	(F) GTGACGGAGAATTAGGGTTC	98	57	IM1∶98	0.998	18.4
(AY846367)	(R) TGTCAGGATTGGGTAATTTG			P1∶93	0.990	
*25S*	(F) GGATTAACGAGATTCCCACT	115	57	IM1∶92	0.996	18.1
(EF463062)	(R) CGGACTAGAGTCAAGCTCAA			P1∶94	0.991	

1 *TUB*, beta tubulin; *CAP*, Capsine phosphatase; *EF1*, Elongation factor 1; *GADPH*, Glyceraldehyde 3-phosphate dehydrogenase; *RUB*, Ribulose-1,5-bisphosphate carboxylase oxygenase; *UBQ*, Ubiquitin; *18S*, ribosomal RNA 18S; *25S*, ribosomal RNA 25S.

2 F, forward primer; R, reverse primer.

3 Values obtained using samplings IM1 or P1 (Table 1).

4 Average Cq value computed over all samples in samplings IM1 and P1.

Specific amplification of each candidate reference gene from cDNA was first tested by PCR followed by electrophoresis on 2% (w/v) agarose gels. Amplicons were sequenced on both strands to confirm the gene targeted was amplified.

### qPCR Experiments

Validation of the candidate reference genes complied with the criteria proposed by [3]. qPCR reactions were as described [4], using the StepOnePlus™ Real-Time PCR System Thermal Cycling Block (Applied Biosystems, Foster City, USA). Amplicon sizes were checked on 3% (w/v) agarose gels. The absence of gDNA contamination was checked for every sample by seeking the intron-containing amplicons of TUB and GAPDH. Assessment of qPCR efficiency was performed using five point, five-fold cDNA dilution series as described elsewhere [9]. To assess candidate reference gene stability, all cDNA samples in samplings IM1 and P1 were diluted 125-fold prior to qPCR. Every diluted cDNA sample was used in two independent qPCRs. As two independent reverse transcriptions had been performed per RNA sample, this yielded four technical replicates per RNA sample.

Data were analyzed with the StepOne Software v2.2 (Applied Biosystems, Foster City, USA). For all genes, the threshold for fluorescence detection was manually set at 0.2 ΔRn, i.e., above the background fluorescence and at the beginning of the region of exponential amplification of the amplification curve.

### Selection of a Set of Reference Genes with Stable Expression Under Herbicide Action in *Lolium sp*

cDNA samples obtained from samplings IM1 and P1 (Table 1) were used to assess the stability of the candidate reference genes. qPCR data were analysed separately for each sampling. The reference genes with the most stable expression were therefore identified independently for each herbicide. This enabled to check whether a same set of reference genes was suitable for both ALS inhibitors. For every candidate reference gene, qPCR data used for stability analysis consisted into the mean quantification cycle (Cq) value computed for the two RT replicates of every RNA sample in each of the two samplings analysed. The Cq values were exported for each sample and each gene after positioning the fluorescence detection threshold manually at 0.2 ΔRn.

The stability of the expression of the candidate reference genes expressed as Cq values was assessed using BestKeeper [19], geNorm [20] and NormFinder [21]. Each of these programs provides a particular statistical framework to estimate the variation in the expression of the candidate reference genes. BestKeeper was used as a first approach. It computes the variation in Cq values and its Standard Deviation (SD) for each gene. Genes with SD <1 are considered stable and ranked on the basis of pairwise correlations between their Cq value and the geometric mean of Cq values of all candidate genes with SD <1 (BestKeeper Index). Candidate genes with the strongest correlations with the BestKeeper Index are considered the most stable.

NormFinder and geNorm require the transformation of Cq values using the 2^−ΔΔCq^ method [20], [21]. NormFinder allows creating subsamples of the sample set, which in this work corresponded to the experimental modalities. NormFinder ranks the candidate reference genes within and among subsamples after their Stability Value (SV) that is based on the variation of their respective transformed Cq values within and among subsamples. Candidate genes with the lowest SVs are considered the most stable. GeNorm computes all possible pairwise variations between the transformed Cq values of the candidate genes. It then provides an estimate of the expression stability (M) of each reference gene tested. M <1.5 identifies candidate genes with a stable expression, and genes with the lowest M value are considered the most stable. As a single reference gene rarely allows adequate normalisation, geNorm also computes the optimal number of genes necessary for reliable normalisation based on the pairwise variation between normalisation factors NF_n_ and NF_n+1_, where n and n+1 are the number of genes considered. NF_n_ is estimated from the geometric mean of the transformed Cq values of the n most stable candidate genes.

### Effect of ALS-inhibiting Herbicides on the Expression Levels of Two Herbicide Target Genes and Five CYPs

*CAP*, *GAPDH* and *UBQ* were used to normalise the expression data of the seven genes investigated. The primers used in qPCR and the expected amplicon sizes for each gene are given in Table 4. qPCR data was generated from samplings IM2 and P2 (Table 2). Expression of the seven genes was compared within each population for each herbicide separately. In each population, gene expression was compared in resistant plants *versus* sensitive plants before treatment (BT) to identify constitutive differences in expression, and in resistant plants *versus* sensitive plants 24 hours after treatment (24HAT), in resistant plants BT *versus* 24HAT, and in sensitive plants BT *versus* 24HAT to identify genes differentially regulated in resistant plants by herbicide application. qPCR results were analysed using REST-MCS version 2 [22] that compares the expression level of a target gene between two groups of samples, taking into account the respective amplification efficiencies of the target gene and of reference genes. Gene expression data consisted into the average Cq values computed from two replicates (two independent qPCR runs, each performed from one of two independent reverse transcription reactions). Gene expression comparison was performed for the seven genes investigated using relative expression ratios computed for each gene and each plant after equation 1 in [23], using three herbicide-sensitive plants in population RG07-063 collected BT as the reference (control) sample.

**Table 4:** Target genes investigated, primer and qPCR information.

Original organism^1^ (accession)	Target gene in *Lolium*sp.^2^ Code (accession,% identity)	Primer sequence (5′-3′)^3^	Ampliconlength (bp)	Tm (°C)	PCR efficiency (%) (regression coefficient)
*Lolium multiflorum*	*ALS*	(F) GCAATCAAGAAGATGCTTGAGAC	132	60	93
(AF310684)	(AF310684, 100%)	(R) TCCTGCCATCACCTTCCATGAT			(0.983)
*Lolium rigidum*	*ACCase*	(F) CACAAGACACAGCTAGATAGTGGCG	174	60	87
(AF359513)	(AF359513, 100%)	(R) TTCCAACAGTTCGTCCAGTCACAAAT			(0.994)
*Lolium rigidum*	*CYP71R4*	(F) GAACATGATGTACCATTTCGACTG	101	60	99
(AB097496)	(AB097496, 100%)	(R) CAGACTTAAGGCCAGGCGACAAC			(0.997)
*Zea mays*	*CYP72A*	(F) CAGTGATGACTTGCTAGGATTG	173	63	97
(AF465265)	(AF321869, 81%)	(R) CATGCTGAGCAGAATTAGTGTC			(0.987)
*Helianthus tuberosus*	*CYP81B1*	(F) GTCTGTTCATGATACCGTTCG	146	63	94
(AJ000477)	(AB159037, 45%)	(R) CTCCGTCATGTCCACCTTC			(0.992)
*Oryza sativa*	CYP81A	(F) AGGGGAGACGGGATGCTGG	179	60	101
(ABC69856)	(HF565053, 78%)	(R) TTGGGCATGGTGATCCCTGG			(0.989)
*Homo sapiens*	*CYP92A*	(F) CTCGATGTTAAGGGGCAGGATTA	176	60	91
(NM_000771)	(HF565054, 26%)	(R) GATCTCCTCCATGTTCAGCTCC			(0.988)

1 Organism from which the gene of interest had originally been isolated.

2 *Lolium* sp. genes investigated in this work. *ALS*, Acetolactate synthase; *ACCase*, Acetyl-coenzyme A carboxylase; *CYP*: cytochrome P450. Accession numbers are given for the *Lolium CYP* genes available in GenBank/EMBL that display the highest amino-acid identity with the *CYP* gene of interest. % identity, % of amino-acid identity between the *CYP* gene of interest and the *Lolium CYP* gene studied.

3 F, forward primer; R, reverse primer.

The genes investigated consisted of *ACCase, ALS* and five *CYP* genes. *ACCase* and *ALS* encode herbicide target enzymes. *ACCase* encodes acetyl-coenzyme a carboxylase, a crucial enzyme in lipid biosynthesis and an herbicide target enzyme [24]. *ACCase* is not the target of the herbicides used herein, and is therefore expected to be stably expressed BT in our experiments. *ALS* encodes the target of the herbicides used herein, and was included in our experiments to investigate whether *ALS* over-expression could play a role in resistance in the populations we analysed.

To identify genes potentially involved in NTSR to ALS inhibitors, we considered six genes encoding CYPs known to have an herbicide-degrading activity. *CYP71R4* had been identified in herbicide-resistant *L. rigidum. CYP71R4* had been shown to catalyse the degradation of the herbicide chlorotoluron that inhibits photosynthesis [25]. *CYP81B1* had been identified in Jerusalem artichoke (*Helianthus tuberosus*). *CYP81B1* had also been shown to catalyse the degradation of the herbicide chlorotoluron [26]. *CYP72A* and *CYP81A6* had been identified in maize and rice, respectively. *CYP72A* and *CYP81A6* had been shown to catalyse the degradation of several pesticides, including herbicides inhibiting ALS [26], [27]. *CYP2C9* is a human gene that had been introduced into transgenic rice plants where it had been shown to catalyse the degradation of chlorsulfuron and imazosulfuron, two herbicides inhibiting ALS [28].

Except *CYP71R4*, all *CYPs* of interest had been identified in plant species different from *Lolium* sp. For each *CYP* gene of interest, primers (Table 4) were designed using the sequence of the *Lolium sp*. *CYP* gene available in GenBank/EMBL that displayed the highest identity in amino-acids with the *CYP* gene considered. The *Lolium* sp. *CYP* gene in GenBank/EMBL most similar to *CYP81B1* had also been reported as *CYP81B1.* The *Lolium* sp. *CYP* genes most similar to *CYP72A*, *CYP81A6* and *CYP2C9* had not been assigned to a *CYP* family. We used a *CYP*-specific Blast tool [29] to identify the family to which each gene could be assigned. To sum up, in this study, *Lolium* sp. genes corresponding to *CYP72A*, *CYP81A6*, *CYP81B1* and *CYP2C9* will be referred to as *CYP72A*, *CYP81A*, *CYP81B1* and *CYP92A,* respectively (Table 4). Amplicons were sequenced on both strands to confirm the amplification of the targeted *Lolium* sp. *CYP* gene.

## Results

### Specificity and Efficiency of Amplification of Candidate Reference Genes

Samplings IM1 and P1 (Table 1) were used to identify reference genes with a stable expression under herbicide action. Each sampling comprised 40 RNA samples (Table 1), and enabled to assess the effect of one herbicide. As two RT reactions were performed per RNA sample, a total of 160 cDNA samples were analysed.

Eight genes were tested as candidate reference genes (Table 3). Agarose gel electrophoresis and the single-peak melting curves obtained for every gene confirmed the absence of primer dimers and of non-specific amplicons (Fig. S1). The absence of gDNA contamination was confirmed by the single-peak melting curves obtained with primers targeting *TUB* and *GAPDH*, which flank an intron-containing region. Specific amplification of the targeted amplicons was confirmed by amplicon sequencing (Table S1).

The non-template controls yielded Cq values that were always above 32 cycles for all primer pairs tested. The corresponding melting curves showed a single small peak that was always located before the position of the single amplicon peak that was obtained in all template samples. As no amplicon peak was detected in the non-template controls, the positive Cq values obtained for non-template controls were considered to be due to primer dimer formation, and were therefore ignored.

qPCR efficiency together with the corresponding correlation coefficients computed from the five-fold dilution series are given in Table 4. Amplification efficiency values obtained for the eight candidate genes were comprised between 90% and 110% and were therefore considered acceptable and comparable [30].

### Stability of Candidate Reference Genes

For each of the two samplings, expression data was subdivided in 10 subsets according to the phenotype (2 subsets), the time after herbicide application (4 subsets) and the *Lolium sp.* population of origin (4 subsets) (Table 1).

#### a) BestKeeper analyses

Gene stability was assessed in each sampling separately using all samples as a single set where all possible combinations of the tested effects (phenotype, time after herbicide application and population) were present. In sampling IM1, all candidate genes were found suitable for normalisation (SD <1 or very close to 1, Table 5). The three most stable genes were by ranking order *GAPDH*, *CAP* and *UBQ.* In sampling P1, five genes were found suitable for normalisation (SD <1 or very close to 1, Table 5). The three most stable genes were by ranking order *GAPDH*, *CAP* and *UBQ*. *18S* and *25S* were the least stable genes in both samplings.

**Table 5:** Ranking of the eight candidate reference genes according to the stability of their expression in samplings IM1 (top) and P1 (bottom) computed using BestKeeper and NormFinder.

Sampling IM1^1^													
Candidate genes^2^	BestKeeper			NormFinder									
				All samples		Phenotype effect		Herbicide effect		Population effect			
						Sensitive/Resistant		Before /2HAT, 6HAT, 24HAT					
	SD^3^	r^4^	rank	SV^5^	rank	SV	rank	SV	rank	SV		rank	
TUB	1.039	0.796*	6	0.723	5	0.199	5	0.463	4	0.237		5	
**UBQ**	**0.870**	**0.863** *	**3**	**0.504**	**3**	**0.151**	**3**	**0.309**	**3**	**0.153**		**3**	
**GAPDH**	**0.565**	**0.857** *	**1**	**0.274**	**1**	**0.103**	**1**	**0.277**	**2**	**0.095**		**1**	
EF1	0.935	0.626*	5	0.672	4	0.181	4	0.484	7	0.211		4	
RUB	0.769	0.310	4	0.889	7	0.269	8	0.463	4	0.263		6	
18S	1.085	0.439*	8	0.974	8	0.261	7	0.477	6	0.316		8	
25S	1.030	0.498*	7	0.864	6	0.247	6	0.502	8	0.277		7	
**CAP**	**0.628**	**0.901** *	**2**	**0.324**	**2**	**0.137**	**2**	**0.221**	**1**	**0.103**		**2**	
**Sampling P1** 1													
**Candidate** **genes** 2	**BestKeeper**			**NormFinder**									
				**All samples**		**Phenotype effect**		**Herbicide effect**			**Population effect**		
						**Sensitive/Resistant**		**Before /2HAT, 6HAT, 24HAT**					
	**SD** 3	**r** 4	**rank**	**SV** 5	**rank**	**SV**	**rank**	**SV**	**rank**		**SV**	**rank**	
TUB	1.053	0.356*	5	0.971	7	0.242	6	0.620	8		0.620	7	
**UBQ**	**0.628**	**0.679** *	**3**	**0.58**	**2**	**0.161**	**2**	**0.385**	**2**		**0.426**	**4**	
**GAPDH**	**0.579**	**0.869** *	**1**	**0.594**	**3**	**0.178**	**3**	**0.455**	**4**		**0.380**	**1**	
EF1	1.143	0.411*	6	0.724	4	0.192	4	0.561	6		0.388	3	
RUB	1.025	0.651*	4	0.784	5	0.239	5	0.381	3		0.521	5	
18S	1.873	0.556*	8	1.273	8	0.314	8	0.602	7		0.684	8	
25S	1.407	0.387*	7	0.941	6	0.241	6	0.541	5		0.554	6	
**CAP**	**0.620**	**0.877** *	**2**	**0.512**	**1**	**0.143**	**1**	**0.337**	**1**		**0.381**	**2**	

1 Sampling composition given in Table 1.

2 The three reference genes selected are in bold. *TUB*, beta tubulin; *CAP*, Capsine phosphatase; *EF1*, Elongation factor 1; *GADPH*, Glyceraldehyde 3-phosphate dehydrogenase; *RUB*, Ribulose-1,5-bisphosphate carboxylase oxygenase; *UBQ*, Ubiquitin; *18S*, ribosomal RNA 18S; *25S*, ribosomal RNA 25S.

3 Standard deviation of Cq values. SD values higher than the threshold value (1.00) are underlined.

4 Pearson’s coefficient of correlation.

5 Stability value.

* associated p-value <0.001.

#### b) NormFinder analyses

This algorithm was first used on each sampling without subset assignment. The most stable genes were by ranking order *GAPDH*, *CAP* and *UBQ* in sampling IM1, and *CAP*, *UBQ* and *GAPDH* in sampling P1 (Table 5). To assess the phenotype effect, every sample in each sampling was assigned to one of the two subsets “sensitive” or “resistant”. The genes most stable between resistant and sensitive plants were by ranking order *GAPDH*, *CAP* and *UBQ* in sampling IM1, and *CAP*, *UBQ* and *GAPDH* in sampling P1 (Table 5).

To assess the effect of time after herbicide application, every sample in each sampling was assigned to one of the four subsets “BT”, “2HAT”, “6HAT” and “24HAT”. The genes most stable under herbicide effect were by ranking order *CAP*, *GAPDH* and *UBQ* in sampling IM1, and *CAP*, *UBQ* and *RUB* in sampling P1 (Table 5). *GAPDH* was ranked the fourth most stable gene under herbicide action in sampling P1.

To assess the population effect, every sample in each sampling was assigned to the corresponding population subset. The genes most stable among populations were by ranking order *GAPDH*, *CAP* and *UBQ* in sampling IM1, and *GAPDH*, *CAP* and *EF1* in sampling P1 (Table 5). *UBQ* was ranked fourth in sampling P1.

Overall, *CAP*, *GAPDH* and *UBQ* were identified as the most stable genes.

#### c) geNorm analyses

**Figure 1:**
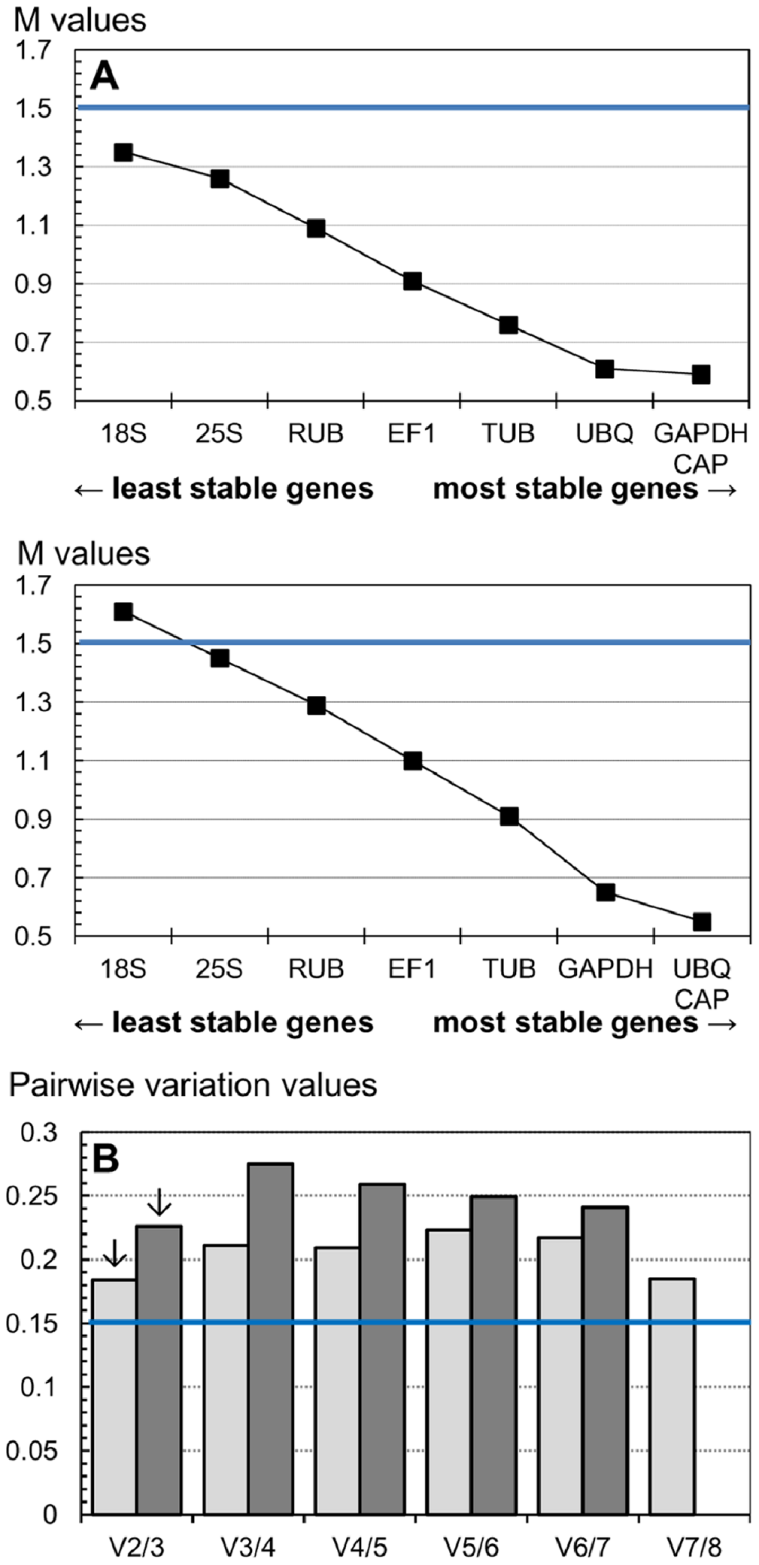
geNorm ranking of the eight candidate reference genes according to their average expression stability value M (A) and determination of the optimal number of reference genes for accurate normalisation (B). A, ranking was performed for all RNA samples in samplings IM1 (top) and P1 (bottom). M-values of the remaining genes at each step during stepwise exclusion of the least stable gene are shown. The genes are ranked according to increasing expression stability (i.e., decreasing M-value). B, pairwise variation analysis to determine the optimal number of reference genes for accurate normalisation in samplings IM1 (open boxes) and P1 (solid boxes). The pairwise variation between consecutive normalisation factors (Vi/i+1) indicating the optimal number of reference genes is arrowed.

Analysis showed that all candidate genes were suitable for normalisation in sampling IM1 (M<1.5). In sampling P1, all genes except *18S* were found suitable for normalisation. *CAP* was identified as the most stable gene in both samplings, with *GAPDH* and *UBQ* being the next two most stable genes. *18S* and *25S* were the least stable genes in both samplings (Fig. 1A).

The optimal number of reference genes required for accurate normalisation was computed in each sampling. *18S* was excluded from the analyses conducted in sampling P1. All values obtained for the pairwise variation between consecutive normalisation factors (Vi/j values, Fig. 1B) were higher than the proposed 0.15 cutoff threshold [20]. As recommended in this case [20], we considered the change in the Vi/j values when including additional reference genes, and we used the lowest Vi/j value to determine the number of reference genes adequate for normalisation. For both samplings, the inclusion of the third most stably expressed gene yielded the lowest variation of the normalisation factor (V2/3; Fig. 1B). Using the three most stable reference genes (*CAP*, *GAPDH* and *UBQ*) as reference genes is considered to be a valid normalisation strategy in this case [20].

### Variation in the Expression of Two Genes Encoding Herbicide Targets and of Five *CYP* Genes

**Figure 2:**
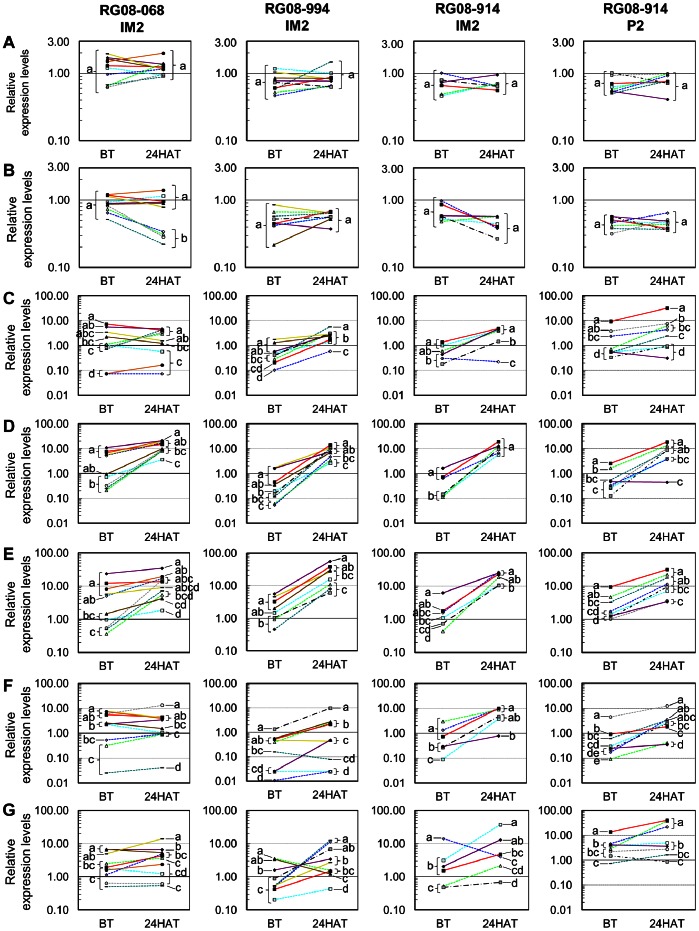
Expression of *ALS*, *ACCase* and five *CYP* genes in individual *Lolium* sp. plants in four groups of samples. A group of samples consists of the plants from the same population that have been used to assess the effect of the same herbicide in sampling IM2 or P2 (Table 2). For every individual plant, the relative expression levels of each gene measured in the absence of herbicide (BT) and 24 hours after herbicide application (24HAT) are connected by a solid line for herbicide-resistant plants, a dotted line for herbicide-sensitive plants or a dashed-and-dotted line for plants with a moderately resistant phenotype. A given colour in all panels in a column (i.e., in a given group of samples) indicates the same individual plant. Panels on lanes A to G, relative expression levels for *ALS*, *ACCase*, *CYP71R4*, *CYP72A*, *CYP81B1*, *CYP81A* and *CYP92A*, respectively. The herbicide considered is indicated below the population code using the sampling code (IM2, iodosulfuron+mesosulfuron; P2, pyroxsulam, see Table 2). Identical letters in a given panel and in a given experimental modality (BT or 24HAT) indicate relative expression levels that are not significantly different.

Samplings IM2 and P2 (Table 2) were analysed using *CAP*, *GAPDH* and *UBQ* for normalisation. Relative expression ratio values ranged from 0.011 (*CYP81A* BT in a sensitive plant in population RG08-994, Fig. 2G) to 56.0 (*CYP81B1* 24HAT in a resistant plant in population RG08-994, Fig. 2D). Overall, considerable variation in the relative expression ratio values was observed among genes, but also among populations, among individual plants in a given population, and among experimental modalities for a given plant (Fig. 2). For instance, *CYP72A* was significantly up-regulated (7.6–fold) in the resistant plants in population RG08-068 compared to the resistant plants in population RG08-994. *CYP72A* was also significantly up-regulated (6.0-fold) in the sensitive plants in population RG08-068 compared to the sensitive plants in population RG08-994 (Figure 2C). Within a given population, substantial differences in relative expression ratios could be observed between plants with a same phenotype (resistant or sensitive). For instance, differences in the relative expression ratios of *CYP71R4* among resistant plants in population RG08-068 could be up to 100-fold (Fig. 2E). As a consequence, no gene showed differences in expression enabling to discriminate all the resistant plants from all the sensitive plants.

No significant differences in the expression of *ALS* were observed between resistant and sensitive plants for all conditions tested (Fig. 2A, Fig. S2A). The highest differences were observed BT in population RG08-068, where the average *ALS* expression in resistant plants was two-fold higher than in sensitive plants. No significant differences in the expression of *ACCase* were observed between resistant and sensitive plants for all conditions tested, except in population RG08-068 in sampling IM2 (Fig. 2B, Fig. S2B). In this population, *ACCase* was found to be significantly up-regulated overall (2.2-fold) in resistant plants compared to sensitive plants 24HAT. This was because *ACCase* expression did not significantly vary 24HAT compared to BT in the resistant plants, while it was significantly down-regulated 24HAT in four of the five sensitive plants in this population (2.4-fold compared to BT) (Fig. 2B, Fig. S2B).

*CYP71R4* was significantly up-regulated BT in the resistant plants in population RG08-068 only (3.3-fold compared to the sensitive plants in this population) (Fig. 2C, Fig. S2C). *CYP71R4* was significantly up-regulated 24HAT in the sensitive plants in population RG08-994 in sampling IM2 and in the resistant plants in population RG08-914 in sampling IM2 (6.4 and 5.8-fold, respectively, compared to the same plants BT).

*CYP72A* was significantly up-regulated BT in resistant plants overall and in populations RG08-068, RG08-994 and RG08-914 in sampling IM2 (8.3, 6.0 and 6.2-fold up-regulated compared to the sensitive plants in the corresponding populations, respectively) (Fig. 2D, Fig. S2D). At 24HAT, no significant up-regulation of *CYP72A* was detected in the resistant plants compared to the sensitive plants in any population. This was due to *CYP72A* being more strongly up-regulated 24HAT compared to BT in the sensitive plants than in the resistant plants in all populations in both samplings (e.g., 15-fold up-regulation on average in the resistant plants in population RG08-994 in sampling IM2 *versus* above 50-fold in the sensitive plants in this population).

*CYP81B1* was significantly up-regulated BT in the resistant plants overall and in each population in sampling IM2 (Fig. 2E, Fig. S2E). It was 8.3, 3.9 and 4.7-fold up-regulated in the resistant plants compared to the sensitive plants in populations RG08-068, RG08-994 and RG08-914 in sampling IM2, respectively. At 24HAT, *CYP81B1* was only significantly up-regulated (4.5-fold) in the resistant plants compared to the sensitive plants in population RG08-994 in sampling IM2. Overall, *CYP81B1* was significantly up-regulated 24HAT in all the plants in populations RG08-994 and RG08-914 and in the sensitive plants in population RG08-068 in sampling IM2, and in the sensitive plants in population RG08-914 in sampling P2 (from 5.5-fold in the sensitive plants in population RG08-994 in sampling P2 up to 15-fold in both the resistant and the sensitive plants in population RG08-994 in sampling IM2, compared to the same plants BT).

*CYP81A* was significantly up-regulated in the resistant plants in populations RG08-068 and RG08-994 in sampling IM2 BT (3.5 and 4.8-fold, respectively, compared to the sensitive plants) and 24HAT (3.9 and 10.5-fold, respectively, compared to the sensitive plants) (Fig. 2F, Fig. S2F).

*CYP92A* was significantly up-regulated BT and 24HAT only in the resistant plants in population RG08-068 in sampling IM2 (11.9 and 6.1-fold compared to the sensitive plants in this population BT and 24HAT, respectively) (Fig. 2G, Fig. S2G). *CYP92A* was also significantly up-regulated BT in the resistant plants in population RG08-914 in sampling P2 (3.6-fold compared to the sensitive plants in this population).

All other differences in expression observed among experimental modalities were not significant. In particular, in the three moderately resistant plants analysed, the relative expression ratios of all the genes investigated were always in range of values observed for sensitive plants (Fig. 2).

Spearman’s rank correlation tests performed on the relative expression ratios of each gene in all resistant *versus* all sensitive plants BT and 24HAT showed a significant correlation between the relative expression ratios of *CYP72A* and *CYP81B1* BT (ρ = 0.83; p<0.001). All other tests were non-significant. When considering the expression data for all five *CYP* genes in each plant individually, it appeared that the plants resistant to (iodosulfuron+mesosulfuron, sampling IM2) generally displayed high relative expression ratio values for most, or all, genes. This was particularly true for two resistant plants in population RG08-068 (Fig. 2C to G). Sensitive plants could also display high relative expression ratio values, but, for a given plant, this was only observed for one or two of the five *CYP* genes. For instance, one sensitive plant in population RG08-068 in sampling IM2 displayed high relative expression ratio values for *CYP72A* (5^th^ and 4^th^ highest expression ratio in population RG08-068 BT and 24HAT, respectively) and *CYP81B*1 (4^th^ and 3^rd^ highest expression ratio in this population BT and 24HAT, respectively), but low relative expression ratio values for the other three genes (8^th^ to 10^th^ highest expression ratios in this population) (Fig. 2C to G). Regarding the two pyroxsulam-resistant plants investigated (sampling P2), one displayed high relative expression ration values for all *CYP* genes except for *CYP81A*. The other displayed relative expression ratio values for all five *CYP* genes that were much lower, and similar to those observed for most sensitive plants (Fig. 2C to G).

## Discussion

### Validation of a Set of Reference Genes Suitable for Gene Expression Studies Under the Action of Herbicides Inhibiting ALS

Eight candidate reference genes were tested using two very heterogeneous samplings (Table 1). As no single method is generally accepted to identify the most stably expressed genes, we used three different algorithms in our analyses. All three identified *CAP*, *GAPDH* and *UBQ* as the genes most stably expressed overall among populations, between phenotypes and under herbicide action for the two herbicides studied. *18S* and *25S* were the genes least stably expressed overall (Fig. 1A and Table 5). The optimal number of reference genes required for adequate expression data normalisation computed by geNorm was three (Fig. 1B). We therefore selected *CAP*, *GAPDH* and *UBQ* as the set of reference genes suitable for normalisation of qPCR data in *Lolium sp.* under herbicide inhibiting ALS stress.

The only other study reporting validated reference genes for the normalisation of qPCR data obtained from plants under herbicide stress considered the grass weed *Alopecurus myosuroides Huds.* and one herbicide inhibiting ACCase. *TUB*, *GAPDH* and *UBQ* were identified as the most stable genes [9]. Our results obtained in a different grass species using two herbicides with a different target enzyme confirmed the stability of expression of *GAPDH* and *UBQ* under herbicide stress. *TUB* was ranked among the three genes most stable under herbicide action in the previous study [9]. Here, *TUB* was ranked among the least stable genes by BestKeeper and NormFinder, and was ranked the fourth most stable gene by geNorm only (Fig. 1A, Table 5). This may be due to the different modes of action of the herbicides used in both studies, which could have a different impact on plant gene regulation. Alternatively, as plants contain several different genes encoding β-tubulin (e.g., [31]), it may be that the *TUB* genes used in each study are not orthologs, and thus do not have the same regulation. From both studies, it appears that *GAPDH* and *UBQ* could be suitable reference genes for the normalisation of qPCR data obtained from grasses under herbicide stress. *CAP* was not tested in the previous study. It was consistently ranked among the two most stable genes in our study. Thus, *CAP*, *GAPDH* and *UBQ* could be suitable reference genes for the normalisation of qPCR data obtained from grasses under herbicide stress.

*Lolium sp*. not being a model species, the reference genes we identified were obtained using the few candidate genes that could be tested based on their stable expression under stress in grasses [4], [14]–[17]. With the rise of next-generation sequencing, genomic resources are now becoming available for a growing number of plant species, including *Lolium sp*. [32]. This will greatly facilitate the identification of genes that are orthologous between closely related model and non-model species. Thus, obtaining a broad range of candidate reference genes for non-model species based on the stability data available for model species will be possible in the near future. In the case of *Lolium sp*., this could result in the identification of yet more stable reference genes for stress response studies.

### Variation in the Expression of *ACCase*, *ALS*, and Five CYP-encoding Genes

The second aim of this study was to investigate potential transcriptional changes of two genes encoding herbicide target enzymes and of five genes encoding CYPs with potential herbicide-degrading activity in herbicide-resistant and sensitive plants in response to the application of herbicides inhibiting ALS.

*ACCase* encodes an enzyme not targeted by the herbicides studied here. As expected, *ACCase* expression did not vary between resistant and sensitive plants or under herbicide action, except in four sensitive plants in one population where *ACCase* was down-regulated after herbicide application. This could be attributed to a redirection of the metabolism of these plants towards the compensation of the herbicide effects [1]. Observing this effect only in one population clearly illustrates the variety of stress responses among populations from a same species.

*ALS* encodes the target enzyme of the herbicides used in this study. Although the resistant plants in population RG08-068 tended to show an up-regulation of *ALS* expression (Fig. 2A), *ALS* expression did not significantly vary between resistant and sensitive plants, or under herbicide action. This is consistent with the literature, where over-expression of the gene encoding the herbicide target had very rarely been reported, and seems an uncommon resistance mechanism in plants [12].

CYPs belong to plant secondary metabolism, and the regulation of their expression is involved in plant response to stresses [33]. Subsets of the *CYP* genes present in a plant genome are involved in different stress response pathways, including herbicide stress response [1]. Among the *CYP* genes involved in the overall response of plants to herbicide stress, a subset can be involved in NTSR. A high expression of the genes in this subset is expected to enhance herbicide degradation in resistant plants compared to sensitive plants [1], [33]. The expression of *CYP* genes involved in NTSR to herbicides can therefore be expected to be up-regulated in resistant plants compared to sensitive plants, either BT and after herbicide application (constitutive up-regulation), or only after herbicide application (herbicide-induced up-regulation) [1], [25], [27], [28]. Thus, comparing the expression of *CYP* genes potentially catalysing herbicide degradation between herbicide-resistant and sensitive plants could allow identification of CYPs involved in NTSR. The expression patterns of each of the five *CYP* genes studied herein varied broadly from plant to plant. Constitutive and herbicide-induced up-regulation was observed both in resistant and in sensitive plants for every gene. No gene displayed expression patterns that were specific to resistant plants. Thus, our data provide no direct proof of the role of any of the five *CYP* genes in NTSR to the two herbicides inhibiting ALS studied here. Two explanations can be proposed.

The first, obvious, explanation is that none of the *CYP* genes investigated is involved in NTSR in the plants studied. Among the five *CYP* genes, only *CYP71R4* had originally been isolated in *Lolium* sp. Yet, its activity on the herbicides investigated here, if any, had not been assessed [25]. The other four *CYP* genes considered had originally been isolated in species different from *Lolium* sp. [26], [27], [28]. The *Lolium* sp. genes studied here are the genes available in GenBank/EMBL that displayed the highest homology with the genes of interest. However, as *Lolium* sp. are not model species, only 50 annotated *CYP* genes have been identified in this genus. In plants, hundreds of *CYP* genes are present that belong to multigenic families, and substrate specificity varies greatly among CYP isoforms [33], [34]. It may be that the genes studied here were not the true homologues of the genes of interest, or that the genes of interest have no homologues with an activity on the herbicides studied in *Lolium* sp. This underlines the need to screen the whole CYP complement (CYPome) of *Lolium* sp. to identify *CYPs* genes involved in NTSR.

A second explanation can be suggested. NTSR had been proposed to evolve by the accumulation of different genes, each conferring a moderate level of resistance. When a combination of genes conferring a high enough resistance level is present in a plant, this plant shows a resistant phenotype [1]. The few studies that investigated the genetic control of NTSR demonstrated that NTSR was governed by a set of genes present in resistant plants. This set varied among plants, even in a given population [35], [36]. The variability in the *CYP* gene expression patterns we observed among plants is consistent with these results. It may thus be that, in the plants studied, some of the *CYP* genes investigated could be involved in NTSR, but none was expressed at a level sufficient to confer NTSR alone (i.e., a combination of several up-regulated genes may be necessary to confer a resistant phenotype). Under this hypothesis, observing similar, high relative expression ratios for a given *CYP* gene in resistant and sensitive plants does not enable to exclude that the CYP in question is not involved in NTSR: additional gene(s) not investigated here can also be present and up-regulated in the resistant plants, but not in the sensitive plants.

Overall, the five *CYP* genes studied were all constitutively up-regulated in resistant plants compared to sensitive plants. Our results thus suggest that at least some secondary metabolism pathways are enhanced in *Lolium* sp. plants resistant to ALS inhibitors compared to sensitive plants, as previously shown in *Arabidopsis thaliana* [37]. Our results are also consistent with a previous study which demonstrated constitutive or herbicide-induced enhanced secondary metabolism and stress oxidative response in *A. myosuroides* plants showing NTSR to herbicides compared to herbicide-sensitive plants [38]. Thus, our study is a further confirmation that NTSR mechanisms belong to a subset of the mechanisms involved in the plant response to herbicide stress [1].

## Conclusions

Using a validated reference gene set to assess the possible role of five *CYP* genes in NTSR to herbicides inhibiting ALS, we illustrated the complexity of investigating the genetic bases of NTSR. NTSR is a quantitative trait, and resistant phenotypes clearly seem to be endowed by several genes. Thus, approaches such as the one conducted herein based on heterologous candidate genes have little chance to be fruitful. As proposed elsewhere [1], an approach enabling to compare the full transcriptome of resistant and sensitive plants appears to be the most straightforward way towards the identification of candidate NTSR genes. In such approaches, the set of reference genes validated herein will be key for the measurement of differences in the expression of candidate NTSR genes identified using transcriptome-scanning methods. Beyond NTSR, our reference gene set can also be used to investigate the genetic bases of the overall response of *Lolium* sp. to herbicide stress. It is very likely that this gene set can also be used in herbicide studies addressing other grass weeds, subject to preliminary validation.

## Supporting Information

Figure S1 Primer specificity test. Melting curves generated for *TUB* (**A**), *CAP* (**B**), *EF1* (**C**), *GAPDH* (**D**), *RUB* (**E**), *UBQ* (**F**), *18S* (**G**), *25S* (**H**), *ALS* (**I**), *ACCase* (**J**), *CYP71R4* (**K**), *CYP72A* (**L**), *CYP81B1* (**M**), *CYP81A* (**N**), *CYP92A* (**O**).
(PPT)

Figure S2 Expression of *ALS*, *ACCase* and five *CYP* genes according to the phenotypes of *Lolium* sp. plants in four groups of samples. A group of samples consists of the plants from the same population that have been used to assess the effect of the same herbicide in sampling IM2 or P2 (Table 2). Red, orange and yellow boxes indicate the plants from populations RG08-068, RG08-994 and RG08-914 in sampling IM2, respectively. Blue boxes indicate the plants from population RG08-914 in sampling P2. Panels **A** to **G**, relative expression levels for *ALS*, *ACCase*, *CYP71R4*, *CYP72A*, *CYP81B1*, *CYP81A* and *CYP92A*, respectively. The relative expression levels of each gene are computed for each group of samples in the resistant plants in the absence of herbicide (BT) using the sensitive plants in the same group as the reference condition (R/S BT), in the resistant plants 24 hours after herbicide application (24HAT) using the sensitive plants in the same group as the reference condition (R/S 24HAT), in the sensitive plants 24HAT using the same plants BT as the reference condition (S BT/24HAT), and in the resistant plants 24HAT using the same plants BT as the reference condition (R BT/24HAT).
(PPT)

Table S1 Sequences of the amplicons obtained in RT-qPCR. Primers binding sites are underlined.
(DOC)
