# Supplementary material for: Reference Genes to Study Herbicide Stress Response in Lolium sp.: Up-Regulation of P450 Genes in Plants Resistant to Acetolactate-Synthase Inhibitors
Source: PLoS One. 2013 May 16;8(5):e63576. doi: 10.1371/journal.pone.0063576 (PMC3656029; doi:10.1371/journal.pone.0063576)
Supplement: Figure S1 — Primer specificity test. Melting curves generated for TUB (A), CAP (B), EF1 (C), GAPDH (D), RUB (E), UBQ (F), 18S (G), 25S (H), ALS (I), ACCase (J), CYP71R4 (K), CYP72A (L), CYP81B1 (M), CYP81A (N), CYP92A (O). (PPT) [file pone.0063576.s001.ppt]

## Slide 1
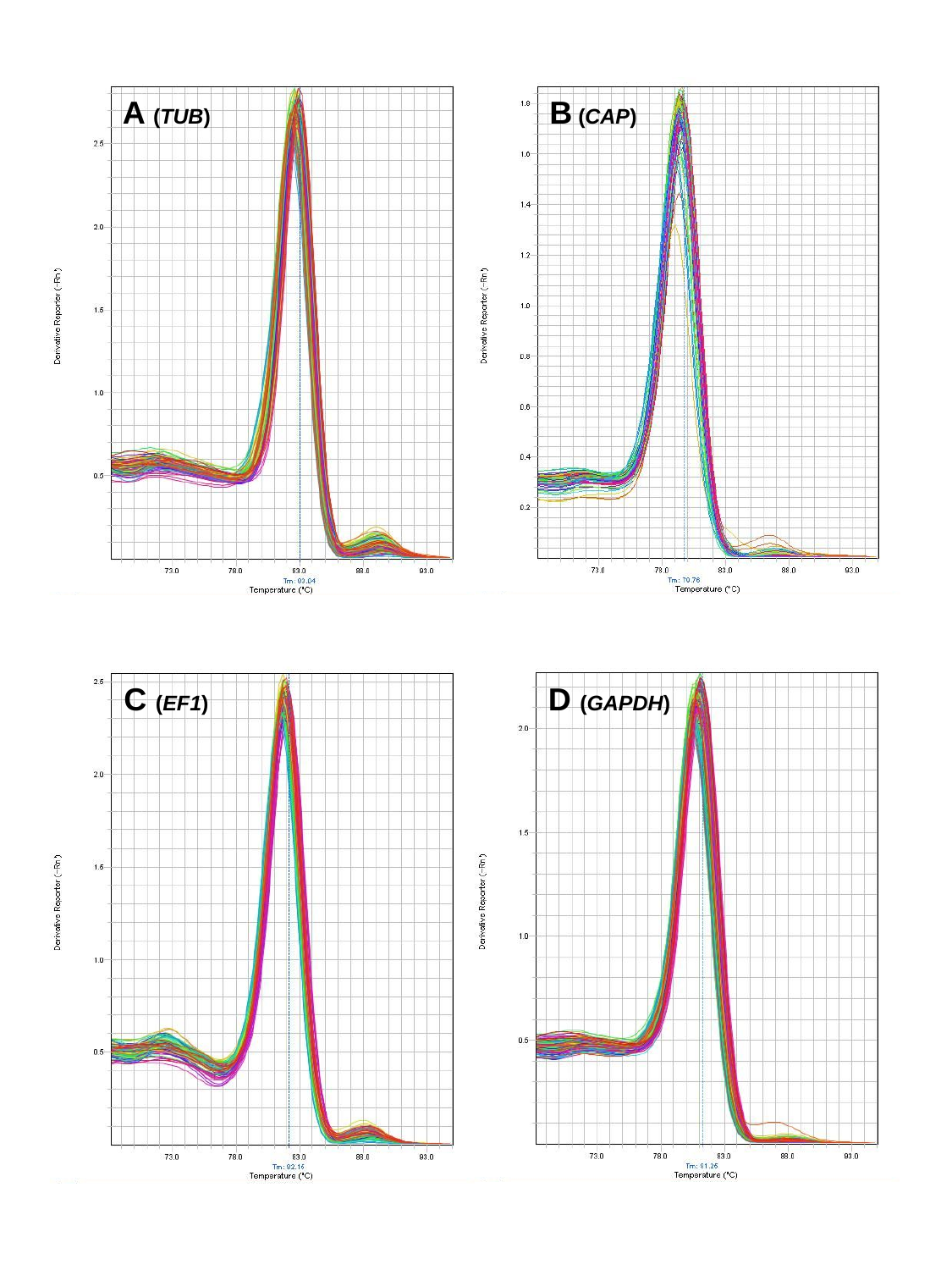

A (TUB)
B (CAP)
C (EF1)
D (GAPDH)

## Slide 2
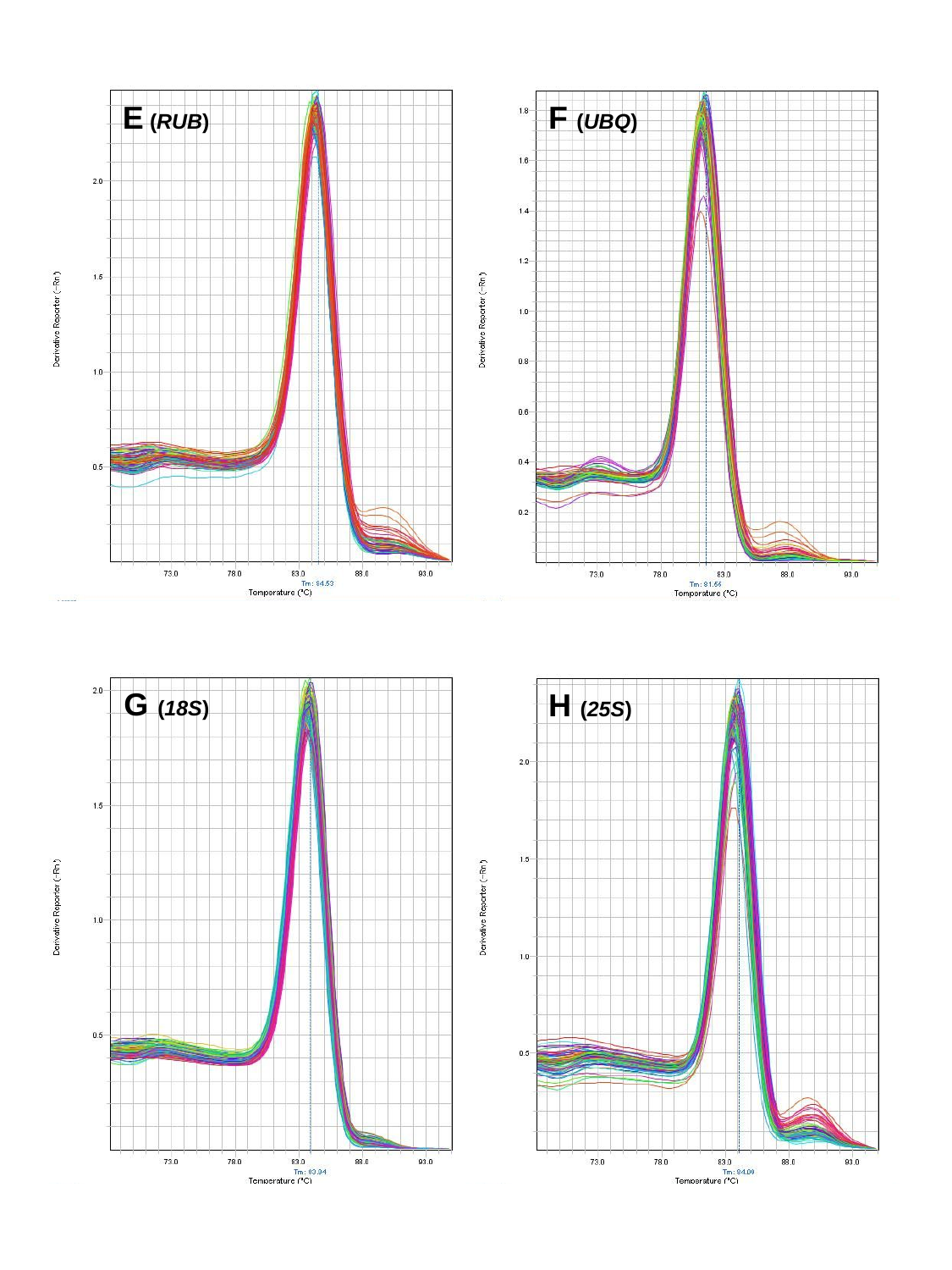

E (RUB)
F (UBQ)
G (18S)
H (25S)

## Slide 3
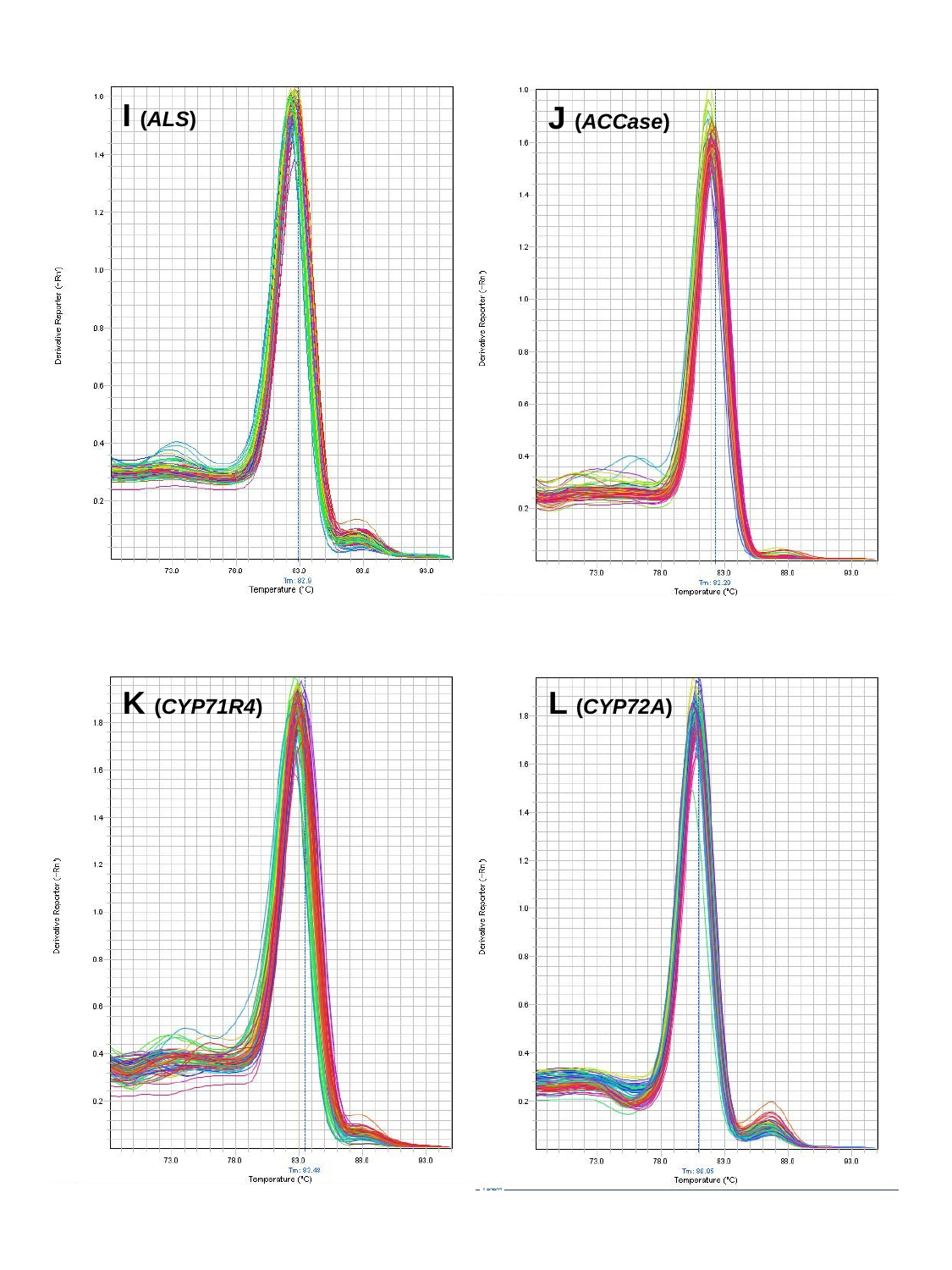

I (ALS)
J (ACCase)
K (CYP71R4)
L (CYP72A)

## Slide 4
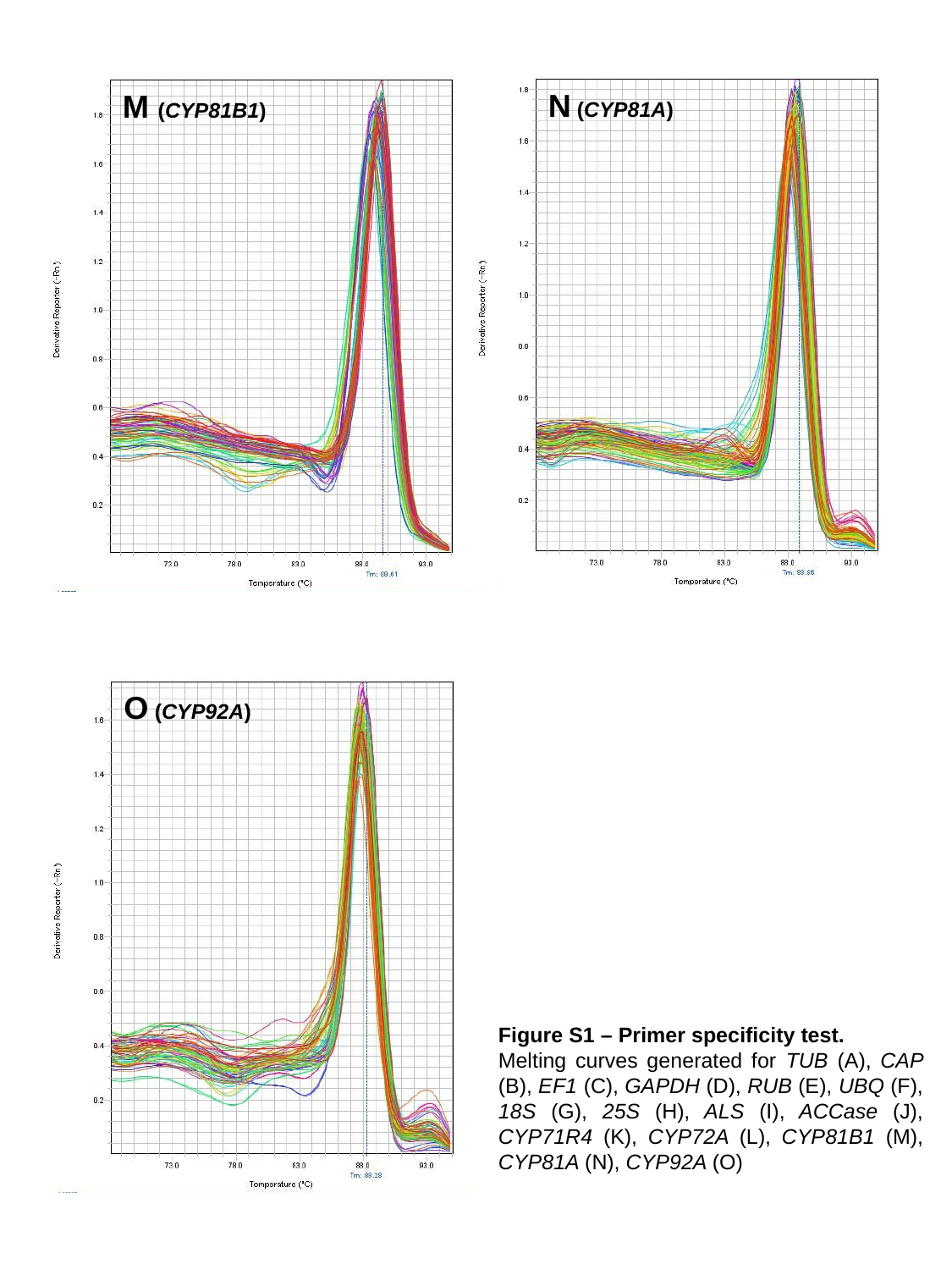

N (CYP81A)
M (CYP81B1)
O (CYP92A)
Figure S1 – Primer specificity test.
Melting curves generated for TUB (A), CAP (B), EF1 (C), GAPDH (D), RUB (E), UBQ (F), 18S (G), 25S (H), ALS (I), ACCase (J), CYP71R4 (K), CYP72A (L), CYP81B1 (M), CYP81A (N), CYP92A (O)
