# Supplementary material for: Reference Genes to Study Herbicide Stress Response in Lolium sp.: Up-Regulation of P450 Genes in Plants Resistant to Acetolactate-Synthase Inhibitors
Source: PLoS One. 2013 May 16;8(5):e63576. doi: 10.1371/journal.pone.0063576 (PMC3656029; doi:10.1371/journal.pone.0063576)
Supplement: Figure S2 — Expression of ALS, ACCase and five CYP genes according to the phenotypes of Lolium sp. plants in four groups of samples. A group of samples consists of the plants from the same population that have been used to assess the effect of the same herbicide in sampling IM2 or P2 (Table 2). Red, orange and yellow boxes indicate the plants from populations RG08-068, RG08-994 and RG08-914 in sampling IM2, respectively. Blue boxes indicate the plants from population RG08-914 in sampling P2. Panels A to G, relative expression levels for ALS, ACCase, CYP71R4, CYP72A, CYP81B1, CYP81A and CYP92A, respectively. The relative expression levels of each gene are computed for each group of samples in the resistant plants in the absence of herbicide (BT) using the sensitive plants in the same group as the reference condition (R/S BT), in the resistant plants 24 hours after herbicide application (24HAT) using the sensitive plants in the same group as the reference condition (R/S 24HAT), in the sensitive plants 24HAT using the same plants BT as the reference condition (S BT/24HAT), and in the resistant plants 24HAT using the same plants BT as the reference condition (R BT/24HAT). (PPT) [file pone.0063576.s002.ppt]

## Slide 1
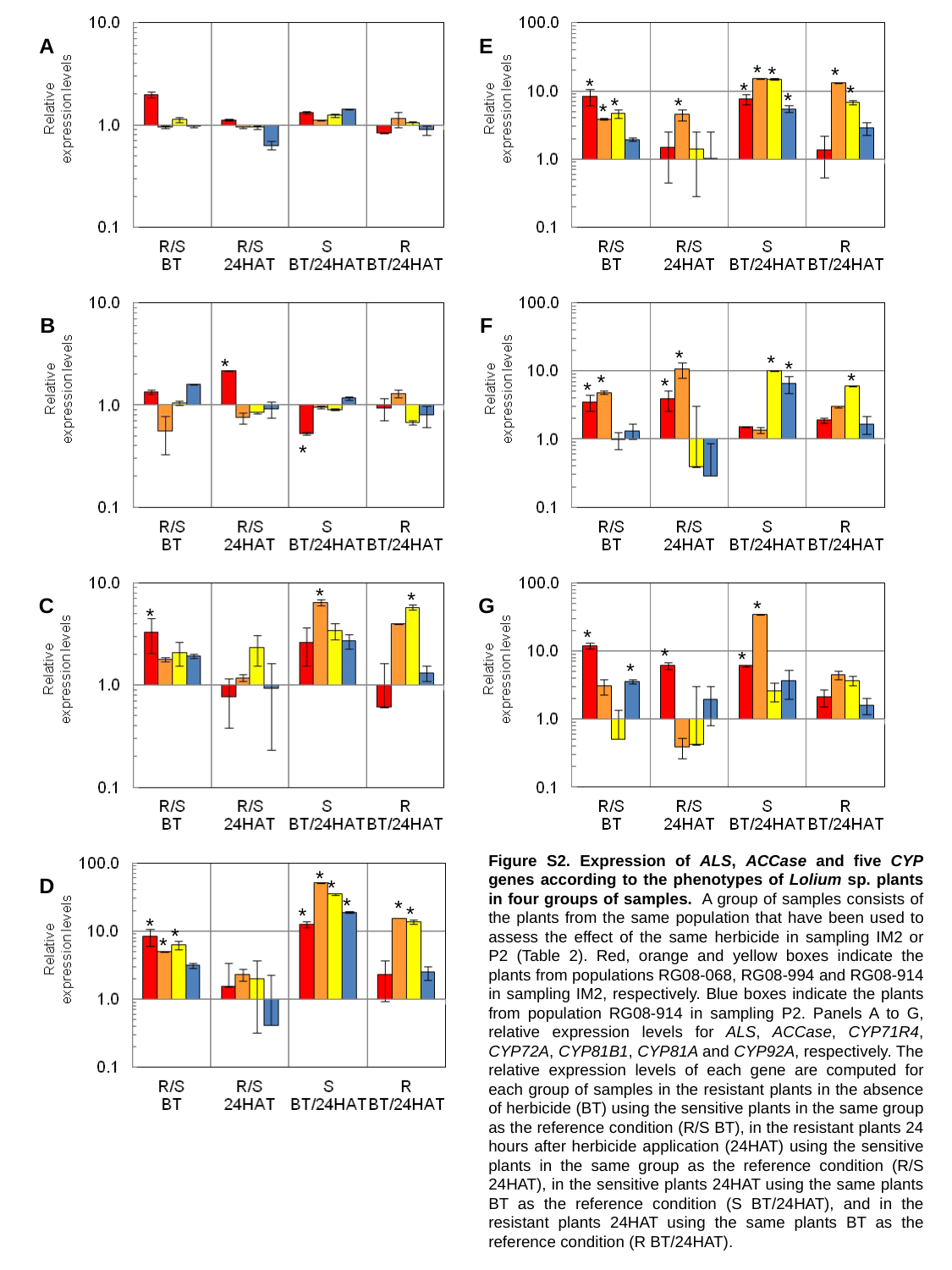

A
E
*
*
*
*
*
*
*
*
*
*
B
F
*
*
*
*
*
*
*
*
*
*
*
C
G
*
*
*
*
*
*
*
D
*
*
*
*
*
*
*
*
Figure S2. Expression of ALS, ACCase and five CYP genes according to the phenotypes of Lolium sp. plants in four groups of samples. A group of samples consists of the plants from the same population that have been used to assess the effect of the same herbicide in sampling IM2 or P2 (Table 2). Red, orange and yellow boxes indicate the plants from populations RG08-068, RG08-994 and RG08-914 in sampling IM2, respectively. Blue boxes indicate the plants from population RG08-914 in sampling P2. Panels A to G, relative expression levels for ALS, ACCase, CYP71R4, CYP72A, CYP81B1, CYP81A and CYP92A, respectively. The relative expression levels of each gene are computed for each group of samples in the resistant plants in the absence of herbicide (BT) using the sensitive plants in the same group as the reference condition (R/S BT), in the resistant plants 24 hours after herbicide application (24HAT) using the sensitive plants in the same group as the reference condition (R/S 24HAT), in the sensitive plants 24HAT using the same plants BT as the reference condition (S BT/24HAT), and in the resistant plants 24HAT using the same plants BT as the reference condition (R BT/24HAT).
