# Supplementary material for: Reference Genes to Study Herbicide Stress Response in Lolium sp.: Up-Regulation of P450 Genes in Plants Resistant to Acetolactate-Synthase Inhibitors
Source: PLoS One. 2013 May 16;8(5):e63576. doi: 10.1371/journal.pone.0063576 (PMC3656029; doi:10.1371/journal.pone.0063576)
Supplement: Table S1 — Sequences of the amplicons obtained in RT-qPCR. Primers binding sites are underlined. (DOC) [file pone.0063576.s003.doc]

**Table S1. Sequences of the amplicons obtained in RT-qPCR.** Primers binding sites are underlined.

>*TUB*

ATACAATGCCACTCTCTCAGTCCACCAGTTGGTTGAGAATGCTGATGAGTGCATGGTTCTTGACAACGAGGCTCTCTATGACATCTGCTTCAGGACTCTCAAGCTGACAACTCCCAGCTTTGGTGATTTGAACCATCTCATCTC

>*CAP*

CTCCAGGGAAGATGCTGAAGATGCAGTCAAATATATTAGTGGCACAATGCTTGATGATCGCCCAATTCGTGTTGATTTTGATTGGGGCTTTCAAG

>*EF1*

CACTGGTCACCTGATCTACAAGCTTGGAGGCATCGACAAGCGTGTGATCGAGAGGTTCGAGAAGGGAGCTGCTGAGATGAACAAGAGGTCCTTCAAGTAC

>*GAPDH*

AGGTTATCAATGACAAGTTTGGCATTGTTGAGGGTTTGATGACCACTGTTCACGCCATGACCGCTACCCAGAAGACTGTTGAT

>*RUB*

GGAGTATGAAACCAAGGATACTGATATCTTGGCAGCATTCCGAGTAACTCCTCAGCCTGGGGTTCCGCCGGAAGAAGCAGGGGCTGCAGTAGCTGCCGAATCTTCTACTGGTACATGGACAAC

>*UBQ*

CAAGAAGAAGACGTACACCAAGCCCAAGAAGCAAAAGCACAAGCACAAGAAGGTGAAGCTCGCCCTCCTCCAGTTCTACAAGGTC

>*18S*

GTGACGGAGAATTAGGGTTCGATTCCGGAGAGGGAGCCTGAGAAACGGCTACCACATCCAAGGAAGGCAGCAGGCGCGCAAATTACCCAATCCTGACA

>*25S*

GGATTAACGAGATTCCCACTGTCCCTGTCTACTATCCAGCGAAACCACAGCCAAGGGAACGGGCTTGGCGGAATCAGCGGGGAAAGAAGACCCTGTTGAGCTTGACTCTAGTCCG

>*ALS*

GCAATCAAGAAGATGCTTGAGACTCCTGGGCCATACTTGTTGGATATCATCGTCCCTCACCAGGAGCATGTGCTGCCTATGATCCCTAGCGGTGGTGCTTTCAAGGACATTATCATGGAAGGTGATGGCAGGA

>*ACCase*

CACAAGACACAGCTAGATAGTGGCGAAATAAGGTGGGTTATCGATTCTGTTGTGGGCAAGGAGGATGGACTAGGTGTGGAGAACATACATGGAAGTGCTGCTATTGCCAGTGCGTATTCTAGGGCATACGAGGAGACATTTACACTTACATTTGTGACTGGACGAACTGTTGGAA

>*CYP71R4*

GAACATGATGTACCATTTCGACTGGAAGCTCCCTAACGGCCAGGACATAGAGTCTTTTGAGCTCATAGAGTCTAGTGGGTTGTCGCCTGGCCTTAAGTCTG

>*CYP72A*

CAGTGATGACTTGCTAGGATTGCTGTTGGAGTCAAATAGACGGGAATCCAATGGGAAAGCAGACCTAGGAATGAGTACCGAAGACATAATTGAGGAATGCAAGCTATTTTACTTTGCAGGTATGGAGACAACATCAGTATTGCTCACATGGACACTAATTCTGCTCAGCATG

>*CYP81B1*

GTCTGTTCATGA

TACCGTTCGGGATGGGGCGGCGGAGGTGCCCCGGGGAGACGCTGGCGCTGCGGACCATCGGAATGGTCCTGGCGACGCTGGTGCAGTGCTTCGACTGGGAACCGGTGGACGGCGTGAAGGTGGACATGACGGAG

>*CYP81A*

AGGGGAGACGGGATGCTGGTGGTACCGTTCGGGATGGGGCGGCGGAGGTGCCCCGGGGAGACGCTGGCGTTGCAGATGGTCGGGATTGTTCTGGCAACGCTGTTGCAGTGCTTCGACTGGGAACGGGTGGACGGCGTGGAGGTGGACATG

ACGGAGGGGCCAGGGATCACCATGCCCAA

>*CYP92A*

CTCGATGTTAAGGGGCAGGATTACGAGCTGCTGCCGTTCGGGTCGGGGCGCAGGATGTGTCCCGGATACAGCCTAGGGCTGAAGGTGATCCAGGTGAGCCTGGCGAACCTACTGCACGGGTTCGAGTGGAAGCTCCCCGACGGCGTGGAGCTGAACATGGAGGAGATC
